# Robust protocol for preparation of EDTA‐Plasma pools for use as quality controls for testing stability of blood biomarkers on the Fujirebio Lumipulse G1200 and Mesoscale Discovery platforms

**DOI:** 10.1002/alz70856_098581

**Published:** 2025-12-24

**Authors:** Batxelli‐Molina Isabelle, Vidal Elisa, Lima‐Guerin Aline, Molaro Damien, Hugo Vanderstichele, Tanja Schubert

**Affiliations:** ^1^ Eurofins ADME Bioanalyses, VERGEZE, Occitanie, France; ^2^ Biomarkable, Gent, Flandre‐Orientale, Belgium

## Abstract

**Background:**

High quality services for biomarkers in a GLP/GCP environment for preclinical and clinical studies require a detailed understanding of the variables that can have an impact on the accuracy and the robustness of the test results. This study will provide details on an experimental protocol to create quality control (QC) samples to support neurobiomarker measurements in clinical studies and to assess their stability in biological samples.

**Method:**

Whole blood was collected from 15 healthy individuals and was processed into EDTA‐plasma within one hour after sample collection. Baseline biomarker concentrations (Aß1‐40, Aß1‐42, pTau217, NF‐L) were measured with the Fujirebio G1200 Lumipulse and the Mesoscale Discovery immunoassay platforms. After testing the fresh samples, aliquots of the samples were stored in low protein binding tubes at ‐80°C for later use. Based on the obtained results, two QC samples were prepared with Low and High concentrations for each biomarker, if needed by spiking the calibrant (< 5.0% of the total volume) into the samples. Using the QC samples, an evaluation was done for each biomarker for a) The biomarker short term stability (i.e. up to 3 freeze/thaw cycles at ‐80°C versus room temperature and bench top stability 2 hours at room temperature) ; b) The long‐term stability after 1 week, 1 month and 3 months of freezing at ‐80°C ; c) The impact of hemolysis and hyperlipemia was tested in parallel.

**Result:**

A concentration range for each biomarker was determined using the individual EDTA‐plasma samples. The mean intra‐ and inter‐ assay variability should be within ± 20%. The concentration in the pooled EDTA‐plasma QC samples was used as a reference for follow‐up studies and should have a deviation within ± 20% from the nominal concentration after stability evaluation.

**Conclusion:**

We have qualified an internal procedure for the preparation of QC samples. We have evaluated the short term and longer‐term stability of EDTA‐plasma for use in biomarker testing for clinical trials.

